# Important Diseases of Small Ruminants in Sub-Saharan Africa: A Review with a Focus on Current Strategies for Treatment and Control in Smallholder Systems

**DOI:** 10.3390/ani15050706

**Published:** 2025-02-28

**Authors:** Peter Kimeli, Kennedy Mwacalimba, Raymond Tiernan, Erik Mijten, Tetiana Miroshnychenko, Barbara Poulsen Nautrup

**Affiliations:** 1 Department of Clinical Studies, Faculty of Veterinary Medicine, University of Nairobi, Nairobi 29053-00625, Kenya; kimeli08@uonbi.ac.ke; 2 Outcomes Research, Zoetis, Parsippany, NJ 07054, USA; kennedy.mwacalimba@zoetis.com; 3 Centre of Excellence, Zoetis, D18 T3Y1 Dublin, Ireland; raymond.tiernan@zoetis.com; 4 Zoetis Belgium S.A., 1930 Zaventem, Belgium; erik.mijten@zoetis.com (E.M.); tetiana.miroshnychenko@zoetis.com (T.M.); 5 EAH-Consulting, 52064 Aachen, Germany

**Keywords:** small ruminants, sheep, goat, sub-Saharan Africa, helminthiasis, viral diseases, bacterial diseases, ectoparasites

## Abstract

Sheep and goats are an important source of livelihood for smallholder farmers in sub-Saharan Africa (SSA). These livestock are almost entirely managed by resource-poor, smallholder farmers and pastoralists. Despite the large number of sheep and goats in SSA, their productivity is low, mainly due to diseases, poor feed, and inferior breeds. This review aims to summarize the most important diseases in small ruminants in SSA, with a focus on current treatment and control strategies. The following diseases were identified as the most significant constraints for small ruminant farmers: helminthoses, including gastrointestinal nematode infestation, lungworm infestation, fasciolosis, and cerebral coenurosis; viral diseases, such as peste des petits ruminants (PPR), sheep and goat pox, and contagious ecthyma (orf); bacterial diseases, including contagious caprine pleuropneumonia (CCPP), pneumonic pasteurellosis, and anthrax; as well as ectoparasite infestations. The diseases have significant economic implications due to mortality and production losses. Depending on the disease, they may also impact trade and export and hinder the introduction of new, more productive breeds. The ability to control diseases more efficiently is often limited due to financial constraints. In the case of infection with internal parasites, a lack of knowledge about the epidemiology of the disease, as well as the availability of appropriate anthelmintics and the development of resistance against commonly used anthelmintics, are often barriers. The control of viral diseases depends on the accessibility, quality, and handling of vaccines, whereas in bacterial diseases, increasing antibiotic resistance and inappropriate antimicrobial treatments pose challenges, as well as the availability of appropriate vaccines and their use. In the case of ectoparasitic infections, a strategic, regular, and appropriate antiparasitic treatment approach is often not achieved.

## Simple Summary

Sheep and goats are an important source of livelihood for smallholder farmers and pastoralists in sub-Saharan Africa (SSA), although their productivity is low, mainly due to diseases, poor feed, and inferior breeds. This review defines and describes economically important diseases affecting small ruminants in SSA and assesses current therapy and control strategies. The following diseases were identified as the most significant constraints for smallholder farmers: gastrointestinal nematode and lungworm infestation, fasciolosis, and cerebral coenurosis; peste des petits ruminants, sheep and goat pox, and contagious ecthyma (orf); contagious caprine pleuropneumonia, pneumonic pasteurellosis, and anthrax; as well as ectoparasite infestations. The ability to efficiently control diseases is often limited due to financial reasons. In the case of infection with internal parasites, a lack of knowledge about the epidemiology of the disease, as well as the limited availability of appropriate, non-resistant anthelmintics, are often barriers. The control of viral diseases depends on the accessibility, quality, and handling of vaccines, whereas in bacterial diseases, increasing antibiotic resistance and inappropriate antimicrobial treatments pose challenges, as well as the availability of appropriate vaccines and their use. In the case of ectoparasitic infections, a strategic, regular, and appropriate antiparasitic treatment approach is often not achieved.

## 1. Introduction

Sheep and goats are an important source of livelihood for smallholder farmers in sub-Saharan Africa (SSA) and are considered a source of food for the household (meat and milk), cash income (when the animal or its products are sold), and social value. Small ruminants are preferred because of their adaptation to harsh environments, reproductive success with a short gestation period, and ability to produce nutritious human food from low-value feedstuff [1]. Small ruminants enjoy access to export markets such as the Middle East, thus contributing to national GDPs [2] and generating hard currency [3].

In 2018, there were 438 million goats and 384 million sheep in SSA [4]. These livestock are almost entirely managed by resource-poor, smallholder farmers and pastoralists [5]. Despite the large number of livestock in SSA, their productivity is low, mainly due to diseases, poor feed, and inferior breeds [6], with diseases and parasite infestations being the most common constraints [7].

This review aims to summarize the most important diseases in small ruminants in SSA, with a focus on current treatment and control strategies. Regarding the diseases of greatest concern, there exists a disparity between community priorities and government priorities, as diseases such as pasteurellosis, coenurosis, and gastrointestinal parasite infestations are of primary concern for smallholder farmers but are often neglected in disease control efforts [8]. A survey was conducted to better understand the main disease constraints in small ruminants in Ethiopia [9]. The project was part of the Consultative Group on International Agricultural Research (CGIAR) Program on Livestock and Fish. The participating farmers identified several diseases and syndromes with local names that affect small ruminants and grouped them into seven major disease categories. Pasteurellosis, contagious caprine pleuropneumonia (CCPP), and coughing were repeatedly mentioned by different local names within the respiratory disease category. Coenurosis was the major disease identified in the group of neurological diseases. Within the skin disease category, sheep and goat pox and orf were mentioned as priority diseases. Diarrhea was reported as a syndrome of gastrointestinal parasite infection. Liver fluke and lungworm were the most important parasites recognized by farmers. Mange mites, ticks, lice, and sheep ked were stated to be common external parasites. Peste des petits ruminants (PPR) and anthrax were considered priority systemic diseases [9]. Coughing was excluded from the list of important diseases because it is a clinical syndrome rather than a disease.

The diseases selected for this review include gastrointestinal nematode infestations, lungworm infections, fasciolosis (liver fluke), and coenurosis, as well as PPR, anthrax, pasteurellosis, CCPP, orf, and goat and sheep pox. These diseases were categorized into four distinct groups for systematic evaluation: helminthoses, viral diseases, bacterial diseases, and ectoparasite infestations.

## 2. Helminthoses

Helminthoses are widespread infections of small ruminants in SSA. Helminths are ubiquitous and many tropical and subtropical environments provide nearly perfect conditions for their survival and development. However, the clinical signs can be less obvious when compared with other livestock diseases. Partly for this reason, infections with gastrointestinal and other helminth parasites are among the most neglected areas of veterinary care in many parts of the developing world, although it has been established that high prevalence rates of infection are associated with poor production and unthriftiness [10].

Nematodes (including gastrointestinal nematodes and lungworms), trematodes (including liver fluke), and cestodes (including *Taenia multiceps*) are the three major classes of parasitic helminths of economic importance affecting goats and sheep in SSA, some of which have zoonotic potential [11].

### 2.1. Gastrointestinal Nematodiasis

Gastrointestinal nematode infections constitute a significant health challenge affecting the productivity and reproductive performance of sheep and goats in SSA [12].

#### 2.1.1. Etiology and Epidemiology

The most common genera of gastrointestinal nematodes in small ruminants in SSA countries are Haemonchus, Trichostrongylus, Oesophagostomum/Chabertia, Trichuris, Teladosargia/Ostertagia, and Nematodirus [13], with Haemonchos contortus, Trichostrongylus colubriformis, and Oesophagostomum columbianum representing the most common gastrointestinal nematodes [11]. However, mixed infections are not unusual [13].

All economically important gastrointestinal parasites of small ruminants have direct life cycles, i.e., they require no intermediate hosts. The mature parasites breed inside the host and lay eggs, which are shed in the feces. After the eggs have passed out, they hatch into first-stage larvae (L1) and molt into second-stage (L2) and the infective third-stage larvae (L3) under appropriate conditions of temperature and humidity, as the larvae need moisture to develop and move. The infective larvae (L3) migrate out of the feces and move up the blades of grass, which the grazing host (sheep or goat) may ingest along with the grass [14]. After ingestion, L3 develops into fourth- and fifth-stage larvae and mature into adults in the abomasum or intestine. However, the third-stage larvae of *Bunostomum* spp. and *Strongyloides* spp. enter the host mainly by skin penetration. After penetration, these parasites are carried in the venous circulation through the heart to the lungs, where they penetrate the alveoli, are coughed up, and then swallowed [11].

Several studies have evaluated the prevalence of gastrointestinal nematodes in sheep and goats in Ethiopia, with estimates ranging from 43.2% to 92.9%. The variation in prevalence is assumed to be due to differences in health management, such as deworming practices and nutrition, stocking density, and co-grazing of ruminants, as well as differences in agroecology, diagnostic techniques used, or the number of animals included in the studies [15]. A meta-analysis estimated the pooled prevalence in Ethiopia at 75.8% [16].

#### 2.1.2. Clinical Signs

Gastrointestinal nematodes can result in anemia due to the hematophagous activities of nematodes, diarrhea due to gastroenteritis or digestion/absorption disruption effects, chronic weight loss, and weakness due to depression of appetite and reduction of feed digestibility [17].

The pathogenesis and clinical symptoms of *H. contortus* are related to the blood-sucking habit of the fourth-stage larvae and adult parasites. Three manifestations occur in goats and sheep: hyper-acute, acute, and chronic haemonchosis. Animals may be found dead without prior signs, with death resulting from blood loss. The disease can be more gradual and accompanied by weight loss, anemia, and hypoproteinemia. The mucosae and conjunctivae of affected animals are extremely pale. More chronic cases show lethargy and muscular weakness, pale mucosae and conjunctivae, and edema, which is often seen under the lower jaw and to a lesser extent along the ventral abdomen. Poor growth in young lambs can result from a reduction in their ewes’ milk production. Susceptibility to haemonchosis varies between breeds and among individuals within a flock. This natural resistance to infection is heritable [18].

Trichostrongylosis is commonly a disease of young animals. The penetration of larvae and adult worms into the intestinal mucosa results in the desquamation of the latter, causing a malabsorption syndrome and hence, protein-losing gastroenteropathy and hypalbuminemia. Heavy infections cause acute enteritis, which is characterized by dark-colored diarrhea and foul-smelling feces. There may be sudden death without evidence of anemia or emaciation, but most commonly, trichostrongylosis presents as a chronic wasting disease characterized by loss of appetite, emaciation, loss of weight, dry skin, diarrhea, edema, and atrophy of skeletal muscles [11].

In young sheep heavily infested with *Osophagostomum*, severe persistent diarrhea may occur, resulting in rapid loss of condition, a hollowed back, stiffness of gait, and elevation of the tail. Anemia is not characteristic and is never marked. Initially, the diarrhea may alternate with constipation, but later, diarrhea persists, with dark and fetid feces [18].

#### 2.1.3. Economic Consequences

Whereas in the developed world, the cost of control has the greatest economic significance, in the developing world, the utmost importance of gastrointestinal nematodes is attributed to productivity losses and mortality. Parasitic gastrointestinal nematode infections account for 28% of the mortality and 8% of weight loss in sheep. Additionally, infected animals produce less milk, meat, and wool of lower quality. The reproductive performance of infected animals is also affected. Annual losses due to gastrointestinal nematodes in Ethiopian meat markets and livestock exports have been estimated at USD 400 million [19].

#### 2.1.4. Treatment, Control, and Prevention

Effective control and management of gastrointestinal nematodes in grazing livestock mainly relies on the strategic use of efficacious anthelmintic drugs. Treatments are generally given during the rainy season, with occasional ad hoc treatments being common among smallholder farmers [5].

In Ethiopia, the most common anthelmintics belong to three families, including macrocyclic lactones (ivermectin), imidazothiazoles (tetramisole and levamisole), and benzimidazoles (albendazole) [20]. However, the consequence of inappropriate anthelmintic treatment procedures, including poor drug quality, improper dosing, and intensive use of a single anthelmintic class, has resulted in the development of resistance to anthelmintic drugs [5]. Five recent studies [13,20,21,22] investigated the resistance status of four commonly used anthelmintics (albendazole, tetramisole, levamisole, and ivermectin) against gastrointestinal nematodes in Ethiopia and Zimbabwe, using the fecal egg count reduction test (FECRT) (Table 1). Before deworming, the three nematode genera found most often in the feces were *Haemonchus* spp., *Trichostrongylus* spp., and *Oesophagostomum* spp. in four of five studies [13,20,21], whereas in one study, *Trichostrongulus* spp., *Haemonchus* spp., and *Teladorsagia* spp. were isolated most frequently [22]. According to the guidelines of the World Association for the Advancement of Veterinary Parasitology, a reduced efficacy, suggesting resistance, is defined as the lower limit of the 95% confidence interval of the FECRT being <90% [23]. According to this definition, resistance was detected against albendazole (five of five studies), tetramisole (one of four studies), levamisole (one of one study), and ivermectin (3 of 5 studies) (Table 1).

Smallholder farmers and pastoralists in Ethiopia use varying parasite control methods, including anthelmintic drugs of different quality and traditional medicines. In Kenya, ethno-veterinary remedies prepared from different plant species are widely used by pastoralists and smallholder farmers [5].

Control of nematode infection in small ruminants also includes proper pasture management [14]. Rotating young animals through pastures ahead of adults may minimize the exposure of young sheep and goats to large numbers of infective larvae. Pasture rotation should follow any administration of anthelmintics [18]. 

### 2.2. Lungworm Infection

Lungworms are important parasitic nematodes of small ruminants that colonize the lower respiratory tract and cause high morbidity, mortality, and economic losses worldwide [24].

#### 2.2.1. Etiology and Epidemiology

The common lungworms in SSA are *Dictyocaulus filaria*, *Protostrongylus rufescens,* and *Muellerius capillaris*. *D. filaria* belongs to the superfamily Trichostrongyloidea, while *P. rufescens* and *M. capillaris* belong to Metastrongyloidea. Although mixed infection may occur, D. filaria is the predominant lungworm in most outbreaks. Two forms of life cycles exist in lungworms of domestic ruminants. One form is the direct life cycle of the Dictyocaulidae, in which the larvae are free-living in their surroundings, i.e., they do not have an intermediate host [10]. In these parasites, the adult females lay larvated eggs in the bronchi that hatch either in the bronchi or after being coughed up and swallowed. The hatched larvae L1 are excreted in feces. On pasture, the larvae molt into the second stage (L2) and develop further into the infective L3. While grazing, animals ingest the infective third-stage larvae (L3). The larvae penetrate the intestinal mucosa and travel to the mesenteric lymph nodes, where they molt into fourth-stage larvae (L4), which arrive in the lungs via lymph and blood. In the lungs, the larvae molt and develop into young adults, which migrate through the bronchial tree as they mature [25]. Pasture infectivity is related to rainfall, which stimulates the activity of the larvae as moisture is essential for their survival and development. Larvae survive best in cool, damp surroundings and can persist for over one year under optimal conditions [10].

Metastrongyloidae are characterized by an indirect life cycle [10]. The first part of the life cycle is similar to that of *D. filaria*. However, after excretion of the L1 in feces, the larvae quickly penetrate snails and develop into infective L3 larvae in a few weeks to several months, strongly depending on weather conditions and snail species. Such infective larvae can survive up to 2 years inside their intermediate host. Livestock become infected after eating contaminated snails or slugs while grazing. L3 larvae are released after digestion. They cross the gut wall and arrive at the lungs through the lymphatic system and the bloodstream [25].

A systematic literature review estimated the pooled prevalence of lungworms in Ethiopia at 40.8% [24].

However, reported prevalence rates of lungworms in Ethiopia varied between 13.4% and 72.4%, depending on differences in nutritional status, level of immunity, and management practices. Additionally, the time and area of study, as well as rainfall, humidity, temperature, and altitude, impacted prevalence rates [25].

#### 2.2.2. Clinical Signs

Lungworm infection is also called verminous bronchitis or verminous pneumonia. It is a chronic and prolonged infection of the lower respiratory tract of sheep and goats, clinically characterized by respiratory distress and pathologically by bronchitis and bronchopneumonia [10]. The most common signs are coughing and unthriftiness [26]. However, symptoms can range from moderate coughing with slightly increased respiratory rates to severe, persistent coughing, respiratory distress, and even death [27]. Generally, only young ruminants in their first grazing season are clinically affected, whereas on farms where the disease is endemic, older animals have acquired strong immunity. Goats appear to be more susceptible to lungworms than sheep. The pathogenic effect of lungworms also depends on their location within the respiratory tract. As *D. filaria* lives in the trachea and bronchi, it affects a large volume of lung tissue, thus being the most pathogenic species, whereas infections with metastrongylidae are more confined to their immediate surroundings [10].

#### 2.2.3. Economic Consequences

Lungworms can have considerable economic repercussions worldwide. Heavy infection with *D. filaria* causes unthriftiness, coughing, weight loss or reduced weight gain, and damage to the respiratory system, which can be fatal [26].

#### 2.2.4. Treatment, Control, and Prevention

Prevention and control can be achieved most effectively by integrating the three interrelated approaches of anthelmintic drug use, improved management practices, and immunization [10,27].

Effective anthelmintics used to treat lungworms include albendazole, levamisole, and ivermectin, which have been licensed for this indication [10,27]. Animals should ideally be dewormed before and after the rainy season [27].

Good management practices, such as the provision of ample nutrition, increase the resistance of the host and, therefore, are important for lungworm control. Larvae of *D. filaria* are not very resistant to dryness but may persist and develop in swampy pastures. Thus, susceptible animals should not be allowed to have access to such areas [10]. In general, grazing young stock in advance of older stock, combined with rotational grazing, avoiding overcrowding, and separating sheep and goat stock, are best management practices to control and prevent lungworm infection [27].

Elimination of the snail intermediate host is an additional important measure for controlling *Metastrongyloidea*. The snails climb plants in the early morning and evening, and animals should not be allowed to graze at those times [10,27].

The most efficient method of preventing verminous pneumonia is to immunize all young sheep and goats. A live vaccine, consisting of larvae attenuated by irradiation, is available. Two doses of the vaccine should be administered to induce a high level of immunity and develop resistance [10]. The vaccine has been used in hundreds of thousands of animals in various countries in Europe and the USA with outstanding success [26]. However, until 2016, there was no report of using a lungworm vaccine in Ethiopia [25].

### 2.3. Fasciolosis

Fasciolosis is globally acknowledged as an important helminthic disease that is classified as a neglected tropical zoonotic disease by the World Health Organization (WHO). The WHO estimates that at least 2.4 million people are infected in more than 75 countries worldwide, with several million people at risk. No continent is free from fasciolosis, and human cases likely exist where animal cases are reported [28].

#### 2.3.1. Etiology and Epidemiology

Fasciolosis, also known as fascioliasis or liver rot, is caused by two trematodes, *Fasciola hepatica* and *Fasciola gigantica*. It is a disease of sheep, goats, and cattle that occasionally affects humans [29]. The two species have a common life cycle: In the vertebrate host, immature eggs are discharged in the biliary ducts and excreted with the stool. The eggs become embryonated in water, releasing miracidia, which invade a suitable snail intermediate host. In the snail, the parasites undergo several developmental stages, the last one being released from the snail, encysting as metacercariae on aquatic vegetation or other surfaces. Mammals acquire the infection by eating vegetation containing metacercariae. After ingestion by the final host, the metacercariae excyst in the duodenum and migrate through the intestinal wall, the peritoneal cavity, and the liver parenchyma into the biliary ducts, where they develop into adults [30]. Humans can become infected by ingesting metacercariae-containing fresh water or water plants or by ingesting food items washed with such water [29,30].

The geographical distribution of *F. hepatica* and *F. gigantica* is determined mainly by the distribution patterns of the snails that act as intermediate hosts. *F. hepatica* is found in temperate areas and high-altitude cooler areas in the tropics and subtropics, whereas *F. gigantica* is predominantly found in the tropics and subtropics [31].

A meta-analysis that evaluated the worldwide prevalence of fasciolosis included 15 studies, reporting prevalence rates in various SSA countries, which were between 3.2% and 60.7% in sheep and 0.4% and 36.0% in goats [32]. A generally higher prevalence in sheep compared to goats is assumed to be associated with differences in grazing behavior between the two species [33].

#### 2.3.2. Clinical Signs

Two phases of fasciolosis are differentiated according to the parasite development: the parenchymal and biliary phases. The parenchymal phase occurs during the migration of flukes through the liver parenchyma and is associated with liver damage and hemorrhage. The biliary phase coincides with parasite residence in the bile ducts and results from the hematophagic activity of the adult flukes and the damage to the bile duct mucosa [29]. Depending on the amount of infectious metacercariae ingested, acute, subacute, or chronic fasciolosis can develop [30]. Acute fasciolosis corresponds to the migratory stages of the life cycle, characterized by extensive damage to the hepatic parenchyma produced by migrating immature flukes after ingesting large numbers of metacercariae (>2000). The clinical signs of acute disease include sudden death, weakness, anemia, and dyspnoea. Subacute fasciolosis is characterized by jaundice, anemia, liver failure, and death within 8–10 weeks [29]. Chronic fasciolosis occurs after ingestion of low doses of metacercariae and results from the presence of mature adult flukes in the bile ducts [29,30]. It is the most common form of liver fluke infection in sheep and goats and occurs when the parasites reach the bile ducts in the liver. The fluke ingests blood, which produces severe anemia, hypoalbuminemia, chronic inflammation, and enlargement of the bile ducts. The clinical signs develop slowly, with affected animals becoming increasingly anemic, losing appetite, and exhibiting pale mucous membranes in the mouth and eyes. Some animals develop edema under the jaw (‘bottle jaw’) [29].

#### 2.3.3. Economic Consequences

The economic losses due to fasciolosis are enormous worldwide and are associated with mortality, morbidity (resulting in reduced growth rate and wool production), condemnation of fluke-infected livers, increased susceptibility to secondary infections, and costs of control measures [30]. In previous studies, the economic losses were estimated at over USD 2 billion per annum to the agricultural sector worldwide, with over 600 million animals infected [34].

In two Ethiopian abattoirs, the annual average losses of condemned livers due to fasciolosis were estimated at USD 3700 and USD 245 for sheep and goats, respectively. However, these losses do not reflect the total costs of fasciolosis, as no other losses or costs were considered [33].

#### 2.3.4. Treatment, Control, and Prevention

Not all anthelmintics are equally effective against all developmental stages of the liver flukes in the body. To treat acute fasciolosis, it is essential to choose a highly effective product against the juveniles that damage the liver parenchyma. For chronic disease, a compound active against adult flukes is required. Triclabendazole is the most commonly used drug due to its high efficacy against adult and juvenile flukes [31]. However, resistance of *F. hepatica* to triclabendazole has been reported [30], and triclabendazole is not available in all countries [29]. Anthelmintics may be used prophylactically to reduce pasture contamination with fluke eggs, during periods of heavy fluke burden or nutritional and pregnancy stress [30].

A supplementary approach to control fasciolosis is reducing snail populations. The best long-term method of reducing mud snail populations is drainage since it ensures the permanent destruction of snail habitats. When snail habitats are limited, a simple control method is to fence off affected areas or treat them annually with a molluscicide [30].

A study conducted in Kenya revealed that 39% of farmers had never dewormed their livestock. The main reason for not deworming was reported to be the cost of dewormers or a general unwillingness to deworm. Most farmers (78.4%) had heard about fasciolosis, but 80% did not know the cause, and 95% were unaware that snails spread liver flukes. Most farmers (77.5%) did not know that fasciolosis is a zoonotic disease [35].

### 2.4. Cerebral Coenurosis

Cerebral coenurosis, caused by the larval stage of *Taenia multiceps*, is often fatal in intermediate hosts, such as small ruminants, and can result in substantial economic losses in livestock farming [36]. Authentic reports of coenurosis began to appear in the literature during the 17th century, although references to nervous disease with the symptoms of coenurosis have been found in texts from the time of Hippocrates [37]. Today, cerebral coenurosis has a worldwide distribution [36]. While the disease has been somewhat controlled in many industrialized countries, it continues to cause a burden in many extensive farming systems [38].

#### 2.4.1. Etiology and Epidemiology

Cerebral coenurosis, also known as gid or stagger, is caused by *Coenurus cerebralis*, the metacestode or larval form of the dog tapeworm *Taenia multiceps* [39]. The adult *T. multiceps* is a parasite of the small intestine of canids such as dogs, foxes, jackals, and coyotes, which act as definitive hosts and are continuous sources of infection through the discharge of eggs in the feces. The intermediate hosts (mainly ungulates such as sheep and goats, as well as humans) acquire the infection by ingesting food or water contaminated with dog feces containing the eggs of *T. multiceps*. Oncospheres released from the eggs in the intestine of the intermediate host reach the CNS, the muscles, and subcutaneous tissue through blood circulation. As the parasite matures, it develops into a large cyst in the brain [37]. The mature cyst measures 5–6 cm in diameter and is filled with a large amount of fluid, containing 400–500 protoscolices. In goats, cysts can also grow outside the CNS, and numerous reports exist describing cysts developing in the musculoskeletal system, in the subcutaneous connective tissue of goats, and in the liver and lungs [36].

The definitive host becomes infected by ingesting the offal of slaughtered animals with a mature *Coenurus* cyst. It has been reported that African smallholder farmers routinely feed their dogs with offal, including sheep and goat heads, even from infected animals [36,40]. In the intestine of definitive hosts, the protoscolices finally develop into adult tapeworms [37]. Proglottids, which can contain more than 30,000 eggs, detach from the end of the worm and pass out in the feces onto pasture where goats and sheep can ingest them [38].

Although dogs are the main definitive hosts, other predators, such as foxes, have also been reported as hosts of this parasite. The presence of cysts in the brain of an infected sheep leads to a thinner skull, making it easier for predators to gain access to the infected CNS [36]. In the rainy season, rain causes the spread of feces of dogs or foxes over grasses, leading to the increased occurrence of the disease during this period [41].

The distribution of coenurosis is worldwide but is most commonly found in developing countries in Southeast Asia and Africa, where sheep and goats are mainly herded over open grazing lands and kept alongside dogs, which help to control the flock. Although outbreaks of acute coenurosis in sheep and goats occur occasionally, the majority of cases are sporadic, chronic infections in animals between one and two years of age [37].

Prevalence rates vary among African countries, being lowest in Ethiopian sheep and goats (4–8%), Kenyan sheep (2.3–4.5%), and Mozambican goats (14.8%). The highest reported prevalence in Africa was 42.1% in Tanzanian sheep and goats [36].

#### 2.4.2. Clinical Symptoms

Cerebral coenurosis can be divided into two phases: the migratory or acute phase and the growth or chronic phase. The early stages of migration through nervous tissue usually pass unnoticed, but in heavy infestations, meningoencephalitis may develop [41], with death occurring within 2–5 days [39]. The chronic form is more frequently reported. Chronic coenurosis typically occurs in sheep aged 16–18 months [41], as a consequence of cyst development in the brain and the associated increased intracranial pressure [39]. The earliest signs are often behavioral, with the affected animal tending to stand apart from the flock and react slowly to external stimuli. As the cysts grow, the clinical signs progress to depression, unilateral blindness, circling, altered head position (head aversion), incoordination, and paralysis [41]. The incubation period varies from 15–33 days for the acute phase and about 6–8 months for the chronic form of the disease [38]. The mortality rate is 100% [39]. Most of the naturally occurring cases of clinical coenurosis in sheep have been observed in young animals, suggesting the existence of age-related resistance to *T. multiceps*. It has been shown that not all exposures to *T. multiceps* result in the development of a mature coenurus, but they do result in the development of immunity [36].

Human infection occurs if eggs are accidentally ingested due to poor personal hygiene. The cysts develop mainly in the brain, spinal cord, or eye, with the most common signs including headache, seizures, paraplegia, hemiplegia, vomiting, and papilledema. However, coenurosis is a relatively rare zoonotic disease [42].

#### 2.4.3. Economic Consequences

Direct losses arising from cerebral coenurosis are due to the death of animals, reduced weight gain, lower live animal prices, and head condemnations, whereas indirect losses arise from treatment attempts [43]. In a survey conducted in Ethiopia, the price of an animal infected with *Coenurus* was less than 50% of the price the farmers can receive for healthy animals [44]. Major economic losses are associated with abattoir brain condemnation in small ruminants destined for export. Though the brain is not commonly consumed in Ethiopia, it is in high demand in Middle Eastern countries [39]. Several studies have evaluated the losses in abattoirs due to head condemnations [45,46,47]; however, these studies do not adequately represent all losses to the community and small ruminant keepers. A survey conducted in two districts in Northern Tanzania estimated the total annual losses in small ruminant households in 2019 at USD 153.83 (Babati District) and USD 1300 (Ngorongo District). The authors attributed these differences to variations in herd characteristics and management practices, such as breed and flock size [43].

#### 2.4.4. Treatment, Control, and Prevention

The control of coenurosis is challenging and has been unsatisfactory to date. Once the clinical syndrome of coenurosis has been established, the prognosis is poor as the outcome is usually death. Although surgical treatment of coenurosis is frequently successful, the use of surgery in animals, especially in small ruminants, is limited and restricted to economically viable and genetically superior and valued animals and is not practiced commonly under field conditions in SSA [36]. A combination of albendazole, fenbendazole, and praziquantel has shown high effectiveness in treating coenurosis [48]. However, treatment is recommended only during the migration stage of the parasite because once the coenurus is fully developed, its rupture after treatment can be very dangerous for the animal [36].

According to a survey conducted in two districts in Northern Tanzania, 38.1% of the small ruminant keepers attempted to treat coenurosis-infected animals with injectable antibiotics, 31.5% did nothing, while 11.3% of the households applied a hot iron to the forehead (area between the horns) of the infected animal. Other control practices reported by respondents include deworming infected goats and sheep, inserting a piece of wood into the nose and scratching gently to encourage nose bleeding, using traditional medicines, and selling or slaughtering diseased animals. According to respondents, none of the above mitigation measures reliably resulted in a curative outcome [43].

There are promising results for a vaccine under development against coenurosis in goats and sheep; however, to date, no commercial vaccines are available. The availability of an effective vaccine in Africa could dramatically impact the prevalence of the disease in goats and sheep in countries where it is known to be endemic [38].

Cerebral coenurosis can be controlled by regular anthelmintic treatment of dogs at 6–8 week intervals, using an effective taenicide, and ensuring correct disposal of sheep and goat brains after slaughter or death of the animal to prevent scavenging by dogs belonging to the general public, which may not receive regular anthelmintic treatment [39]. However, for successful control of coenurosis, community knowledge of the epidemiology of the disease needs to be improved [49]. In the survey, conducted in two districts in Northern Tanzania, over 99% of the respondents were able to describe clinical signs and postmortem findings of coenurosis, although fewer than 2% were aware of appropriate control measures for the condition. A large proportion (>86%) did not know the cause of coenurosis, and about 84% and 90% of the respondents did not know the role of dogs in the transmission of *Taenia multiceps*. Although 65.9% of respondents who kept dogs reported deworming them, they used anthelmintics for small ruminants (albendazole, levamisole, and ivermectin), which are likely ineffective against cestodes. All dogs had access to the brains of slaughtered/dead sheep and goats, either directly (thrown to dogs) or indirectly (discarded in the bush or buried in shallow pits) [43]. Furthermore, farmers also contribute significantly to environmental contamination with the parasite by opening the skulls of diseased sheep out of curiosity or to establish their own diagnosis [36].

### 2.5. Stilesiosis

This section would not be complete without the mention of Stilesiosis, which is a condition caused by *Stilesia* spp., which typically affects the liver and bile ducts (for species like *Stilesia hepatica*) or the intestines (for species like *Stilesia globipunctata*) in small ruminants, especially sheep and goats [50]. Limited information is available on its epidemiology (thus, it is not included in the summary tables); however, *S. hepatica* is more frequently reported and is widely distributed across sub-Saharan Africa, particularly in warm and humid regions [51,52]. The transmission of *Stilesia* spp. occurs when animals ingest intermediate hosts (like oribatid mites) while grazing, completing the life cycle of the parasite [50].

Large numbers of *S. hepatica* are often found in the bile ducts of sheep and goats at slaughter, and liver condemnations are a source of considerable economic losses on aesthetic grounds. Parasite-affected livers may show signs of mild cirrhosis with thickening of bile ducts. Heavy infestations are frequently seen in apparently healthy sheep and goats, with complete occlusions of the bile ducts. In central Ethiopia, the prevalence of *S. hepatica* in both sheep and goats was determined to be 53.1%. The study estimated an annual financial loss attributed to the parasite at the abattoir to be USD 5807.45 [50]. In a different study on the Kenyan coast, liver condemnations due to *S. hepatica* infestation were estimated at USD 14,807 between 1989 and 2004 in Taita Taveta district [52]. Overall, S. hepatica accounted for 79% and 84% losses in total liver condemnations in slaughtered goats and sheep, respectively. Some studies show that praziquantel is effective against parasites, but there is a need for further research on available therapeutic options against *Stilesia* spp. [53].

## 3. Viral Diseases

### 3.1. Peste Des Petits Ruminants

Peste des petits ruminants (PPR) or sheep and goat plague is a contagious disease mainly affecting small ruminants [54]. It is considered to be the most significant economic threat to the development of sustainable sheep and goat production across the developing world, particularly in Africa and Asia [55]. More than 80% of the global 2.5 billion small ruminants are at risk of PPR [56]. PPR is a transboundary animal disease [57] and a listed disease by the World Organization for Animal Health (WOAH) [58]. After the successful global eradication of rinderpest in 2015, the Food and Agriculture Organization of the United Nations (FAO) and the WOAH (formerly OIE) have targeted PPR as the next global eradication goal [59].

The disease was first identified in the early 1940s in the Ivory Coast. It has since spread to many countries in Africa, the Middle East, and Asia, resulting in a significant geographical expansion of the disease over the last 15 years. Today, PPR is endemic in large parts of Africa, Asia, and the Near and Middle East [60]. As of November 2021, 59 countries, plus one zone of Namibia, were officially recognized as “PPR-free”. However, 138 countries did not have this status, including 67 countries with recent evidence of PPR infection [56].

#### 3.1.1. Etiology and Epidemiology

PPR is caused by the small ruminant morbillivirus (commonly known as PPR virus), a member of the genus *Morbillivirus*, in the family *Paramyxoviridae*. The PPR virus exists as one serotype with four genetically distinct lineages [61], which are generally correlated with the geographical distribution of the virus [62]. It is closely related to other genus members, including the rinderpest virus, measles virus, and canine distemper virus [61].

PPR virus affects all small ruminants, but goats are much more susceptible than sheep [54]. Cattle, camels, and several wild ruminants have been infected occasionally; however, there is no evidence to show that the disease is maintained in these populations without concurrent infection in sheep and goats [55].

PPR virus is transmitted primarily through direct contact between infected and susceptible animals. Therefore, communal grazing areas and live animal markets are important places for the spread of the virus. Large amounts of infective virus are excreted in secretions and discharges from the eyes, nose, and mouth, as well as in feces, and the primary route of infection is respiratory [63]. Indirect infection via fomites or water and feed troughs is possible but is thought to occur at a very low level. [62] The virus does not survive long outside a host [55].

The pooled prevalence of PPR in Africa was estimated in a meta-analysis at 40.16% and 41.79% in sheep and goats, respectively. The difference between sheep and goats was not statistically significant [55].

#### 3.1.2. Clinical Signs

The incubation period of PPR is typically 4–6 days but may range from 3 to 10 days [60]. The super-acute form is frequently observed in goats and especially in newborn kids, and leads to sudden death (occurring within a few hours), with the main signs being severe hyperthermia and congested mucous membranes. The acute form is the classical form of PPR, and symptoms are identical to those observed in the super-acute form but evolve over a longer period. Nasal discharge becomes mucopurulent and obstructs the nostrils, and erosive and ulcerative lesions are observed on the gums, tongue, inner side of the cheeks, palate, and even the larynx. The tongue is covered with a foul-smelling whitish coating. Respiratory signs, such as increased frequency and cough, are present [54]. Gastroenteritis and severe diarrhea are also commonly observed [64]. Two evolutions are possible: death in eight to ten days or healing with lifelong immunity. The sub-acute form is less severe and less frequently observed. In this form, the disease progresses over ten to fifteen days with inconstant clinical signs and does not result in mortality [54].

In susceptible populations, the morbidity can reach 90–100%. Mortality rates vary but can reach 50–100%. Morbidity and mortality rates are lower in endemic areas and among adult animals than in young animals [60].

#### 3.1.3. Economic Consequences

The economic impact on food security and livelihoods makes PPR one of the most important diseases for people relying on small ruminants for income, with losses from outbreaks disproportionately affecting poor, rural households [65]. The economic importance of PPR has attracted the attention of the global community, and consequently, PPR has been targeted by the international community for worldwide eradication by 2030 [59].

The household income losses were calculated in a bioeconomic model for 15 SSA countries, ranging between 0.6% and 44.8% of the total annual income. Households in lower income quintiles were relatively more severely affected by PPR than households in upper quintiles. As expected, the greater the dependence on small ruminant production for household income, the greater the impact [54].

Moreover, PPR affects the national and international movement and trade of sheep, goats, and their products [55,66].

#### 3.1.4. Treatment, Control, and Prevention

There is no specific treatment against the disease [62]. Symptomatic animals are treated with antibiotics and vitamins. Although not recommended by the WOAH, the practice of giving antibiotics to affected animals is widespread among herders and livestock owners [67], and oxytetracycline has been proven to be effective in achieving recovery from clinical signs [64].

Efficacious attenuated PPR vaccines are available, providing strong immunity [60]. The international initiative for the control and eradication of PPR by 2030, launched by the WOAH and the FAO, relies on vaccination campaigns and disease surveillance [59]. The strategy consists of four necessary steps: 1. assessment, 2. control, 3. eradication, and 4. post-eradication follow-up. In stage 2, mass vaccination (100%) of all animals older than 3 months of age is suggested in the first phase, followed by a phase of targeted vaccination of animals between 4 and 12 months of age [67]. In 2022, over 60% of the planned 1.5 billion vaccine doses to be used in the mass vaccination phase of the PPR eradication program were deployed [56].

### 3.2. Sheep and Goat Pox

Sheep pox (SP) and goat pox (GP) are among the most important diseases of sheep and goats in many African countries, following PPR and contagious caprine pleuropneumonia [68]. SP was likely present in Asia and Europe as early as the second century [69]. Today, both viruses are endemic in Africa north of the Equator, the Middle East, and Asia [70]. SP and GP are transboundary animal diseases [57] and listed diseases by the WOAH [58].

#### 3.2.1. Etiology and Epidemiology

*Sheeppox* virus (SPV) and *goatpox* virus (GPV) are the causative agents of the corresponding diseases. Strains of SPV and GPV can pass between sheep and goats, although most strains cause more severe clinical disease in only one species [70]. Generally, the diseases are less commonly seen in indigenous breeds than in exotic breeds. SP and GP are not zoonotic, and wild ungulates have not been identified as reservoirs for the diseases [71].

SPV and GPV transmission mainly occurs through direct contact, indirect contact with infected objects or fomites, and insects, which can mechanically transmit the viruses [72]. However, the movement of infected animals is the primary mode of transmission. Outbreaks occur during all months of the year but are more frequent during the cold or wet seasons [69].

In endemic regions of Ethiopia, an overall prevalence was calculated at 14.5% in sheep and 15.69% in goats [73]. Similar prevalences (17% in sheep and 14% in goats) were reported for another Ethiopian region [68]. Large-sized flocks were more likely to be seropositive than small-sized flocks [73], and young animals were more likely to be positive than older ones. [68,73].

#### 3.2.2. Clinical Signs

The incubation period of sheep pox and goat pox is between 8 and 13 days [70].

Some breeds of European sheep may die of acute infection before developing skin lesions. In other breeds, there is an initial rise in rectal temperature followed by the development of macules and papules, which may cover the body or be restricted to the groin, axilla, and perineum. Within 24 h of the appearance of generalized papules, affected animals develop rhinitis, conjunctivitis, and enlargement of all the superficial lymph nodes. As the papules on the mucous membranes of the eyes and nose ulcerate, the discharge becomes mucopurulent, and the mucosae of the mouth, anus, and prepuce or vagina become necrotic. Breathing may become labored and noisy due to developing lung lesions. If the affected animal does not die in this acute phase of the disease, the necrotic papules form scabs, which persist for up to 6 weeks, leaving small scars [70]. Skin lesions can be detected on the entire body of infected animals but can be easily seen in hairless areas [3], and they are susceptible to fly strikes [70]. The development of secondary pneumonia is common, and abortion may occur [69].

The clinical signs vary considerably with the breed of the host and the strain of capripoxvirus. Indigenous breeds are less susceptible and commonly show only a few lesions. However, lambs that have lost their maternally derived immunity, animals that have been kept isolated, and animals brought into endemic areas from isolated villages, particularly if they have been subjected to the stress of moving long distances and mixing with other sheep and goats, can often develop a generalized and sometimes fatal infection [70].

Morbidity varies between 10% and 85%. Mortality usually ranges from 5% to 10% in local breeds [3], but can reach up to 100% in fully susceptible animals [70].

#### 3.2.3. Economic Consequences

SP and GP are major constraints to the introduction of exotic breeds of sheep and goats to endemic areas and to the development of intensive livestock production [70]. The diseases can affect trade, import, and export. SP and GP are economically important due to production losses, such as decreased weight gain and milk yield, damage to wool and hides, increased abortion rates, and increased susceptibility to other diseases, while also being a direct cause of death [71]. The mean weight loss due to SP and GP has been estimated at 15% in sheep and goats, compared with non-affected animals. In the same study, the costs for replacing dead animals were 61.1% and 50.0% higher than the payments the farmers received for selling their diseased sheep and goats, respectively [74]. In a study in Ethiopia, the highest losses from SP and GP were due to mortality and abortion, whereas the least losses were calculated for treatment [75].

An integrated stochastic production and economic herd model was developed for transhumance and sedentary herds. Losses at herd level were between 60.24 GBP and 1778.01 GBP, depending on the assumed severity of the outbreak (severely or slightly affected), considering herd sizes of 7 and 38 sheep and 12 and 23 goats in sedentary and transhumance herds, respectively. In scenario analyses, vaccination was economically viable for all subsidy levels in high-risk areas. Vaccinating all animals in low-risk areas only realized benefits with 100% government subsidies [76].

#### 3.2.4. Treatment, Control, and Prevention

There is no effective treatment for SP and GP infection. Broad-spectrum antibiotics are indicated to control secondary bacterial infections. Topical treatment is important for skin lesions [77].

In most countries in which SP and GP are enzootic, a slaughter policy would be impracticable, and movement controls would be impossible to enforce. In these countries, vaccination and the implementation of biosecurity measures are considered the only suitable control measures. The most feasible, economical, and viable method is the implementation of a mass vaccination program [77].

Live attenuated vaccines are safe and effective in controlling the diseases. In Ethiopia, approximately 22 different types of vaccines have been produced for domestic use and export markets. Among others, the live attenuated (KSGPV) O-180 vaccine strain has been manufactured, which has been demonstrated to be a safe, effective, and affordable method for control. However, the safety and effectiveness of the vaccine depend on proper storage, transportation, and handling, and the absence of adequate infrastructure could hinder the implementation of sufficient herd immunity [3].

Vaccine failures have occurred, which could be associated with insufficient coverage and vaccine quality. In response, the National Veterinary Institute in Ethiopia has been working extensively to improve the current KSGP vaccines. Awareness campaigns for farmers have been recommended to promote vaccine management and handling [3]. In Nigeria, however, no official vaccination control program exists and commercial vaccines are not readily available [74,76].

Inactivated vaccines are also available but may only provide short-term immunity [78], and have not yet proven to be practical under field conditions [71].

### 3.3. Contagious Ecthyma (Orf)

Contagious ecthyma, also known as orf, is a debilitating and economically important disease and a zoonosis [79]. It has been reported since 1920 in sheep and goats in many parts of the world [80], and it is endemic in most livestock herds [81].

#### 3.3.1. Etiology and Epidemiology

Contagious ecthyma is caused by the orf virus, a member of the genus *Parapoxvirus* in the subfamily *Chordopoxvirinae* and family *Poxviridae.* It affects sheep, goats, and their wild relatives [82]. The orf virus is mainly transmitted through direct contact or by exposure to fomites carrying the virus [83]. The virus can be found in skin and mucosal lesions, including scabs, and is thought to enter the skin through cuts and abrasions. Infected offspring can transmit the virus to their dam’s teats when nursing. The virus is reported to remain viable on wool and hides for approximately a month after the lesions have healed. Orf virus is very resistant to inactivation in the environment, and it has been recovered from dried crusts for several months or years in the laboratory. However, survival of the virus may be shorter in wet conditions [82].

Humans acquire the infection from contact with infected or recently vaccinated animals and/or fomites in conjunction with skin trauma [83]. Person-to-person transmission has been reported in very rare instances [82].

A study conducted in Northwest Ethiopia found an overall prevalence of 12%, with sheep being affected more often (15.5%) than goats (8.5%). Higher prevalence of orf was recorded for animals with poor body condition and in animals between 1 and 2 years of age [84].

#### 3.3.2. Clinical Signs

The disease initially presents itself as papules (elevation of the skin) that progress to blisters (fluid-filled pouches) or pustules before encrusting in the skin of the lips. They can spread around the outside and inside of the mouth, on the face, lips, ears, vulva, lets, scrotum, teats, the interdigital region, and eyes. Extensive lesions on the feet can lead to lameness, and lesions on the udder, which are due to direct contamination during nursing, can cause mastitis in does and ewes [83].

In flocks where the disease occurs for the first time, morbidity rates can be up to 70%. Mortality, however, is usually low (<1%), although increased rates (up to 90%) have been reported in lambs after secondary bacterial infections. The disease is most dangerous in young animals which may refuse to nurse and can die of starvation [83]. Stressors such as concurrent illnesses, malnutrition, or poor management can increase the severity of the clinical signs [82].

#### 3.3.3. Economic Consequences

The economic importance of orf is usually associated with poor growth in lambs, mastitis in ewes, and death in worst-case scenarios. A reduced growth rate or weight loss may be considerable due to a reluctance to suckle or graze. Losses will depend upon the severity of the infection and within-flock prevalence. The impact varies from country to country, both qualitatively and quantitatively. A significant impact will be seen in severe outbreaks where mortality may be considerable. The presence of contagious ecthyma in a country limits the trade of new breeds and the development of intensive animal production [83].

#### 3.3.4. Treatment, Control, and Prevention

Since orf is a viral disease, there is no specific treatment. Few traditional herbal therapies have shown some effectiveness in SSA. Recently, several antiviral drugs, such as Cidofovir, have been found to be highly effective in treating complicated orf virus infections in sheep, goats, and human beings [85]. However, none of the drugs tested are licensed for use in sheep and goats. Antibiotics are usually unnecessary except in severe cases, but the topical application of antibiotic formulations or antiseptics may be appropriate. If lambs have obvious difficulty suckling, bottle feeding may be necessary to prevent severe weight loss and debilitation [83].

Live attenuated virus and attenuated tissue culture vaccines are widely used in the fight against orf. However, conventional attenuated vaccines have drawbacks, as there is a potential risk of virulence reversion and spread of the virus, and infections in humans have been reported [86]. Accordingly, vaccination with live virus vaccines should not be recommended on a farm with no previous history of orf outbreaks as the live virus may contaminate the environment [85].

## 4. Bacterial Diseases

### 4.1. Contagious Caprine Pleuropneumonia

Contagious caprine pleuropneumonia (CCPP) is considered one of the most severe and highly infectious diseases in goats. The disease causes significant economic losses for countries involved in goat farming, particularly those in Africa [87]. CCPP was first described in 1873 in Algeria. Today, CCPP affects goats in more than 40 countries, including many SSA countries [88]. CCPP is a listed disease by the World Organization for Animal Health [58].

#### 4.1.1. Etiology and Epidemiology

CCPP is caused by *Mycoplasma capricolum* subspecies *capripneumoniae* (*Mccp*). Goats are considered naturally susceptible domestic species. Although *Mccp* and antibodies have been found in clinically affected and healthy sheep [89], the exact role of sheep in the maintenance and spread of *Mccp* to goats needs to be further investigated [90]. Wild reservoir species may include wild ruminants. However, their role as reservoirs or dead-end hosts for this disease is unclear [91]. CCPP is not a zoonotic infection [89].

Transmission of CCPP occurs through aerosol droplets from infected or carrier animals to healthy animals. Factors such as overcrowding, especially during confinement in night enclosures, stress due to extreme weather, lack of vaccination against *Mccp*, poor management, and concurrent infections contribute to the occurrence and spread of the disease. Where extensive and traditional husbandry dominates, pathogens spread when animals meet at watering points and grazing areas. Infective *Mccp* is believed to persist in chronic, latent carrier goats that have recovered from clinical CCPP, although isolation efforts have not been successful. These animals are considered to be responsible for the perpetuation of the disease in endemic areas [88].

A pooled prevalence of CCPP has been estimated in meta-analysis to be 23.21% in African goats, with a high variability between different studies [92]. The variability in the prevalence and incidence of CCPP is reflected by local epidemiological conditions and the diagnostic tests employed [91]. Additionally, differences in husbandry practices, vaccination history, and sample sizes contribute to the high variability in prevalence rates in different studies [90]. The main studies included in the meta-analysis were conducted in Ethiopia, and prevalence rates varied between 1.96% and 48.3% in goats [92].

The meta-analysis also revealed the relevant prevalence of CCPP in sheep in Africa, with a pooled estimate being calculated at 24.12% and thus not statistically different from that of goats. This finding was further supported by other studies, where sheep kept with goats were found to be seropositive in almost all study areas [90].

#### 4.1.2. Clinical Signs

The incubation period generally lasts 10 days but may vary between 2 and 28 days [88]. CCPP is characterized by respiratory symptoms, and the disease may manifest as a peracute, acute, or chronic form. Peracute and acute forms usually result when fully susceptible herds are exposed to the pathogen, whereas chronic forms occur in endemically affected areas. In peracute forms, death usually occurs within 24–72 h and, by definition, without respiratory signs [91]. The acute form is characterized by unilateral sero-fibrinous pleuropneumonia with severe pleural effusion [89], resulting in anorexia, weakness, exercise intolerance, high fever (41–43 °C), and respiratory signs, including dyspnea, polypnea, coughing, and nasal discharge. Respiration becomes painful, and affected animals stand with limbs abducted and their necks extended. In terminal stages, the goats are unable to move, and death follows quickly. Abortion can occur. In sub-acute or chronic forms, signs are milder, with coughing noticeable following exercise [88].

In an area where CCPP is endemic, the disease usually develops only in naive animals, whereas in areas free of disease, a large population can be affected after the introduction of the pathogen, resulting in high morbidity (up to 100%) and mortality (80–100%) [91].

#### 4.1.3. Economic Consequences

CCPP is considered one of the most severe and highly infectious diseases of goats. It causes heavy economic losses to countries involved in goat farming, especially in Africa, Asia, and the Middle East [91]. It is estimated that the total yearly cost of CCPP is about USD 507 million in endemic areas, leading to major economic losses. Economic losses are due to morbidity, mortality, and a decline or loss of production performance, in addition to costs involved in prevention, control, and treatment [91], with the main losses being attributed to death, abortion, and milk and weight loss [2]. Diseased animals are usually culled in developed countries, which can be challenging in resource-constrained settings [91].

In a study conducted in Tanzania, mortality contributed the highest percentage of economic loss (62%), followed by abortion losses. Treatment costs were estimated at 5% of total losses because most cases were treated locally without consultation with veterinary professionals [2].

#### 4.1.4. Treatment, Control, and Prevention

For technical as well as financial reasons good-quality vaccines are not widely used in all regions where CCPP is highly prevalent. Therefore, goat owners frequently rely on antibiotic treatments in case of an outbreak. Antibiotics can stop morbidity and mortality and reduce the spread of *Mccp*. However, treated animals may become potential carriers [88]. Several antibiotics have been used to treat CCPP. However, macrolides, especially tylosin, are considered the drug of choice against *Mccp*. The use of oxytetracycline has proven effective and has been used for quite a long time, increasing the risk of side effects (teratogenicity in goat kids) and antibiotic resistance. Also, combinations of dihydrostreptomycin sulfate, with or without penicillin G procaine, have been used for the treatment of CCPP. Other antibiotics like fluoroquinolone (enrofloxacin, danofloxacin), aminoglycosides (streptomycin), and pleuromutilin (tiamulin) are being used, and newer ones among these classes are being explored against *Mccp*. However, costs, availability, handling, and the risk of creating a carrier state are major determinants affecting the application of antibiotics in CCPP-prevalent areas. Currently, *Mccp* strains are susceptible to a wide range of antibiotic classes. However, some strains resistant to tetracyclines, erythromycin, or streptomycin have been identified, indicating the possibility of increasing antibiotic resistance in the future [91].

In Tanzania, most cases were treated locally without consultation of veterinary professionals, with farmers tending to use low dosages of antibiotics because they were cheaper compared to higher doses typically used by professionals. This allowed them to save money on quality medicine, longer-duration dosage administration, and consultation fees for veterinary personnel [2].

Vaccination is the most cost-effective technique for the control of CCPP [88]. However, few CCPP vaccines are manufactured in Africa and other countries. Concerns have been raised regarding the quality of these vaccines [91]. An inactivated vaccine with saponin as an adjuvant was produced in Kenya, which has been shown to reduce morbidity and mortality significantly in goats with CCPP. However, post-vaccinal reactions associated with the use of saponin have been reported. A new inactivated vaccine has been developed at the National Veterinary Institute in Ethiopia, which could be used for mass vaccination. At present, a key problem regarding the production and widespread use of vaccination is the lack of technological advances that would enable the production and packaging of sufficient doses of the purified protein vaccine [88].

### 4.2. Pneumonic Pasteurellosis

Pneumonic pasteurellosis, also known as respiratory mannheimiosis, is one of the most economically important infectious diseases of ruminants and has a wide prevalence across continents [93]. Although *Pasteurella* sp. are distributed worldwide, the microorganisms are reported most frequently in Asia and Africa [94].

#### 4.2.1. Etiology and Epidemiology

Multiple bacterial, viral agents, and other stressors can be involved in pneumonic pasteurellosis, although *Pasteurella* species, with *Mannheimia haemolytica*, *Pasteurella multocida*, and *Bibersteinia trehalosi,* are the most important agents. The bacteria were formerly grouped under the genus *Pasteurella* but were subject to intensive revision and reclassification [95]. *P. multocida* has 16 serotypes classified using lipopolysaccharide antigens and five serogroups (A, B, D, E, F) based on capsular antigens. *M. haemolytica* is composed of a collection of 17 serotypes distinguished by capsular antigens. Globally, the 17 *M. haemolytica* serotypes were reorganized into *Bibersteinia trehalosi* containing four serotypes, *M. haemolytica* containing 12 serotypes, and *M. glucosida* with one serotype. Despite the 17 serotypes, additional untypable isolates have been recognized, which account for more than 10–15% of the clinical isolates from ruminants. Serotypes have demonstrated varying degrees of pathogenicity and virulence, and serotype associations with specific host species are noted. Immunity is serotype-specific, with few or no cross-reactions between serotypes [96]. The bacteria are common commensals of the tonsils and nasopharyngeal microflora of healthy sheep and goats. Small ruminants contract pneumonic pasteurellosis due to exposure to stress factors or unfavorable environmental conditions. Stress may be either psychological, resulting from fear, restraint, or rough handling, or physical, such as extremely hot or cold weather with high levels of humidity, overcrowding in a limited space, poor ventilation, feed and water shortages, and distant transport or shipping. Other stressful situations, such as a high burden of internal or external parasites and the mixing of animals from different sources, can also be involved [95]. The disease is transmitted through inhalation of infected nasal secretions and droplets, coughed up or exhaled by infected animals or recovered carriers in which the infection persists in the upper respiratory tract [94]. *Pasteurella* species are extremely sensitive to the environment. However, when conditions are appropriate, the disease can spread quickly and infect a large percentage of the flock within hours, especially when animals are closely confined [97].

In Ethiopia, overall *Pasteurella* species were isolated in 25% of apparently healthy sheep from lungs or nasal cavities, with the majority (87.5%) being *M. haemolytica* [98]. In another study in Ethiopia, all sheep and goats randomly sampled were serologically positive for at least one serotype, of which 92.45% had antibodies against four or more serotypes [96].

#### 4.2.2. Clinical Signs

A wide variety of clinical signs, ranging from sudden death to occasional coughing, may occur in sheep affected by pneumonic pasteurellosis. Clinical manifestations of acute respiratory distress can develop in adult animals up to 14 days after being exposed to stress, but a much earlier onset is more typical. Infected animals appear extremely dull with reduced appetite and notable depression, high fever, coughing, dyspnoea, muco-purulent nasal discharge, and anorexia. Later, a productive cough can develop, accentuated by physical effort or movement. Marked dyspnea with an expiratory grunt may be observed in the advanced stages of the disease. Young animals are more susceptible than adults and develop more severe infections in which sudden death may occur with or without any previous clinical signs [95]. Both morbidity and case-fatality rates vary between 50% and 100%. Morbidity depends on the immune status of the herd, either acquired naturally or induced by vaccination [94].

#### 4.2.3. Economic Consequences

In Ethiopia, pneumonic pasteurellosis has been a major challenge to veterinary practitioners [94]. It is a high-priority disease causing significant economic losses through high mortality and morbidity, high treatment costs, reduced weight gain, delayed marketing, and unthriftiness [97].

#### 4.2.4. Treatment, Control, and Prevention

Antimicrobial drugs represent the most powerful tools for controlling such infections. However, increasing rates of antimicrobial resistance may markedly reduce the efficacy of the antimicrobials used to control Pasteurella and Mannheimia infections [96]. Different studies [98,99,100] investigated the resistance rates of *M. haemolytica* and *P. multocida* strains. In these studies, neither *M. haemolytica* nor *P. multocida* was resistant (0.0%) to Chloramphenicol. Resistance rates for Penicillin-G were between 50% and 89.3% (*M. haemolytica*) and between 66.7% and 75% (*P. multocida*). In one study, both *Pasteurella* sp. were 100% resistant to Vancomycin. All results are included in Table 2 and Table 3.

Vaccination is the most effective control strategy to reduce the incidence and burden of the disease and to minimize antimicrobial use. Currently, several vaccine types exist against pasteurellosis globally. Problems with vaccination arise in areas with more than one serotype circulating due to the lack of cross-protection. In Ethiopia, for example, only a monovalent *P. multocida* serotype vaccine was commercially available, although *P. multocida* serotypes A and D and 11 serotypes belonging to *M. haemolytica* have long been detected. Consequently, repeated outbreaks were reported in Ethiopia, even among vaccinated sheep and goats, which practitioners and communities attribute to vaccine failure [96].

### 4.3. Anthrax

Anthrax is a serious zoonosis with a global distribution that affects livestock, wildlife, and humans. It is one of the earliest zoonoses described in ancient literature and is believed to have its roots in SSA [101]. Today, anthrax is enzootic in many African and Asian countries but has also been reported in some countries in the Americas and Europe [102].

#### 4.3.1. Etiology and Epidemiology

Anthrax is caused by a Gram-positive, endospore-forming bacterium named *Bacillus anthracis* [103]. The bacterium has a characteristic bimodal lifestyle, including a spore (in the environment) and a vegetative form (inside the host). Spores develop on exposure to air [102]. They are dormant in the environment and can persist in the soil for several years, triggering outbreaks under favorable climate conditions [104]. Domestic and wild herbivores are most susceptible, whereas carnivores and omnivores are relatively resistant [102].

Soil is the primary reservoir for anthrax spores [102]. The initial source of anthrax typically appears when the soils of previously buried cases of anthrax are disturbed [101]. The spores enter the body via ingestion, inhalation, or penetration through broken skin [104]. When the spores enter a mammalian host, they germinate to produce vegetative bacteria, which rapidly divide and encode for the capsule and virulence factors, such as the anthrax toxins. The dividing bacteria require the toxins to evade the host immune system and disseminate systemically, normally through regional lymph nodes associated with the site of infection. The bacteria multiply to extremely high numbers in the bloodstream, where the production of the anthrax toxins leads to lethality [105]. During the terminal stage of the disease, death occurs because of toxic shock with severe pulmonary dysfunction and cardiac failure. It is believed that the toxins produce cytokine-storm-like symptoms resulting in anaphylactic shock [102]. Goats are less frequently affected than sheep, and this might be associated with different browsing behaviors [103].

Anthrax is endemic to Africa, with numerous outbreaks across several SSA countries [106]. Sporadic cases are reported in SSA yearly [103,107]. In addition, large-scale outbreaks can occur, as reported in 2023, when five countries in East and Southern Africa suffered from anthrax outbreaks with more than 1100 suspected human cases [108]. In humans, outbreaks of anthrax are usually due to the consumption of infected meat, contact with contaminated carcasses, animal products, including hides, hair, and infected animal body fluids, or contaminated utensils [102]. Animal owners in resource-limited settings are usually at high risk of contracting anthrax infection because of their animal handling practices [103].

#### 4.3.2. Clinical Signs

The incubation period is usually 3–7 days. Peracute forms are common with rapid disease progression. Animals are often found dead without any prior clinical signs. The dying animals are usually found bloated without rigor mortis or incomplete rigor mortis, and the absence of blood clotting is the most prominent characteristic of anthrax. The blood may fail to clot, and blood discharges can occur from the nostrils, eyes, mouth, and anus after death. Acute forms may manifest with fever, congested and sometimes hemorrhagic mucous membranes, muscle spasms and labored breathing, dysentery, and diarrhea with terminal convulsions and death [104].

#### 4.3.3. Economic Consequences

The agricultural sector suffers substantial losses due to anthrax outbreaks in livestock. The high mortality rates impose substantial financial burdens on farmers and livestock owners. Furthermore, the fear of anthrax contamination disrupts trade and the export of livestock and their products, hindering economic growth and adversely affecting livelihoods [101].

A novel Outbreak Costing Tool, developed by the US Centers for Disease Control and Prevention, estimated the costs of investigating and responding to an anthrax outbreak in Tanzania with 81 suspected human cases and 16 cattle deaths (December 2018 to January 2019). The total costs for investigating and responding to this outbreak were estimated at USD 102,232, and travel and transport were identified as the highest cost category, followed by medical countermeasures [109].

#### 4.3.4. Treatment, Control, and Prevention

The anthrax bacterium remains susceptible to many antibiotics, such as penicillin, oxytetracycline, amoxicillin, chloramphenicol, ciprofloxacin, doxycycline, erythromycin, gentamicin, and sulfonamides. However, penicillin and oxytetracycline have been proven to be the most suitable options in field conditions. Fever is present in the early stages of the disease before other signs are noticeable. During that time, the animal responds well to proper treatment. Hyperimmune anthrax serum has been suggested with antibiotic therapy. However, the antiserum is not usually produced or used for treatment in animals [104].

As the toxin is the cause of death, the importance of early administration of antibiotics still needs to be stressed [110]. Even if bacteremia is cleared after a certain critical stage of infection, i.e., once the toxins have entered cells in sufficient quantity, the toxins manifest their effects [105]. Therefore, antibiotics may clear the infection if administered too late but still fail to save the patient or animal [110].

The eradication of anthrax might not be a realistic aim, owing to the persistence of anthrax spores in the environment. However, anthrax can be prevented. Vaccination of animals helps to prevent the spread of the disease to humans and protect individuals at high risk. Furthermore, education and awareness campaigns are crucial to informing communities about the risk, transmission pathways, and preventive measures to curb anthrax, which includes proper disposal of infected carcasses [101]. Post-mortem examinations are not recommended because of the risk of infection and the exposure of the vegetative organisms to air, which triggers the formation of endospores and, hence, contamination of the environment [11].

Livestock anthrax vaccines are available in almost all countries that experience outbreaks or sporadic cases on an annual basis [110]. Despite the availability of locally produced vaccines, livestock vaccination is often neglected. This, combined with the practice of slaughtering, butchering, and distributing meat from animals that were either sick or had succumbed to anthrax, contributes to an increased likelihood of anthrax in SSA [101].

## 5. Ectoparasite Infestations

Ectoparasites are not associated with high mortality rates in small ruminants but are important causes of unthriftiness and production losses in affected animals [11]. They are among the major factors contributing to the shortage of high-quality hides and skins, as they cause serious skin defects that result in downgrading of quality and rejection [111]. Moreover, they are important vectors of protozoan, bacterial, viral, and rickettsial diseases [112]. Ectoparasites are a major problem where favorable ecological conditions coincide with poor husbandry [113].

### 5.1. Etiology and Epidemiology

Newly introduced animals are a major source of infection for a flock. However, ectoparasite infestations also commonly occur at watering points, during movement, and in communal grazing settings [112].

Several studies have investigated the prevalence of ectoparasites in sheep and goats in different areas of Ethiopia. The prevalence of ectoparasites varied widely, between 47.7% [114] and 94.62% [112] in sheep and between 34.9% [115] and 91.86% [112] in goats. Some studies also estimated the prevalence separately for different ectoparasites. In most studies, ticks, lice, and fleas were the most common ectoparasites. An overview of the percentage distribution of ectoparasites from five studies conducted in different areas of Ethiopia is displayed in Table 4.

The association between ectoparasites and poor body condition has been described inconsistently across various studies. Poor body condition can lower the immune response of the animal, making it more susceptible to infestation, and/or it may be a consequence of chronic ectoparasite infestation [111].

### 5.2. Clinical Signs

Ectoparasites can cause a wide range of health problems, such as mechanical tissue damage, irritation, inflammation, hypersensitivity, abscesses, weight loss, lameness, anemia, and, in severe cases, death of infested animals [114].

In cases of flea infestations, animals show unthriftiness, restlessness, pruritus, alopecia, body weakness, scratching, rubbing, and licking. Severe flea infestations can cause anemia in young animals [11]. Ticks are blood-sucking ectoparasites. Tick bites may damage the host at the site of attachment, causing local injury and predisposing it to secondary bacterial infection [118]. Severe lameness has been noted when ticks attach around the coronary band [11]. In addition to their known significance in transmitting various diseases, some species can cause paralysis or toxicosis [118]. The lesions caused by louse infestations are almost circular and of small size, and the extent of damage to the leather depends on the presence or absence of secondary infection [117]. Mange commonly presents as discomfort and scratching-related skin conditions that lead to irritation, exudation, crusts, and scabs forming on the skin. However, owners usually become aware of mite infestations only when the disease has progressed to the point of irreversible skin damage [113].

Sheep keds are blood-sucking parasites, and heavy infestations may cause severe anemia, which can weaken the animal and make it more susceptible to other diseases [111]. *Melophagus ovinus*, the common sheep ked, causes a defect commonly seen in sheep skins and known as ‘cockle’ [117].

The resulting skin defects can be directly related to the parasite’s blood-sucking habit and/or to self-inflicted secondary damage [112].

### 5.3. Economic Consequences

Hide and skins from small ruminants are important agricultural products. The export of hides and skins is an important source of income and foreign exchange for many countries. Ethiopia has considerable potential to produce substantial quantities of hides and skins, but their quality is very low. About 35% of sheep and 56% of goat skins are rejected due to ectoparasite damage, and about one-quarter to one-third of all skins processed at tanneries are unsuitable for export. As a result, ectoparasitic infestations cause serious economic losses to smallholder farmers, the tanning industry, and the country as a whole through animal mortality, decreased production, downgrading, and rejection of skins and hides. Most tanneries state that only 10 to 15% of harvested skins qualify for top grades. The estimated economic loss due to reduced sheep and goat skin quality in Ethiopia is around USD 14 million per year [118].

### 5.4. Treatment, Control, and Prevention

Commonly, there are three classes of compounds available for the treatment and prevention of external parasitic infestation: organophosphates (e.g., diazinon), synthetic pyrethroids (e.g., flumethrin and high-cis-cypermethrin), and macrocyclic lactones (e.g., ivermectin and doramectin) [118].

Ticks are treated and controlled with acaricides only where ticks are present in large numbers, using sprays, dips, or pour-on solutions. In cases of lice infestation, spraying or dipping with an acaricide is effective and should always be done twice. For sheep keds, spraying or dipping with an insecticide after shearing will also destroy the flies. The treatment and control of mites are similar for all species, involving dipping with an acaricide or administering parenteral ivermectin. However, most ectoparasite infestations can be prevented and controlled through environmental control [118].

Field treatment practices do not always follow recommendations. In pastoral districts of northeastern Ethiopia, where control campaigns using diazinon were implemented once a year, the prevalence of ectoparasites remained extremely high, with 94.6% and 91.9% in sheep and goats, respectively. The irregular application campaigns did not change the burden of ectoparasite infestation. Factors that might be responsible for the remaining high prevalence of ectoparasites in these areas include the limited effectiveness of the diazinon in use, the method of application (spray rather than dipping), animal husbandry, production system (e.g., mixing of flocks during communal browsing/grazing, at watering points, marketplaces, vaccination posts, and elsewhere), the nature of the ectoparasite, as well as the absence of environmental control. For this reason, some pastoralists continued to rely on traditional treatments, such as washing animals with plant-based solutions. In total, in the study area, 20% of the pastoralists never used acaroids for treatment but relied instead on traditional methods [112]. However, in another study conducted in Ethiopia, most goat owners used modern therapeutic agents, such as ivermectin and acaricides, to treat mange mites, whereas only a few owners used ethnomedicines, which was attributed to the provision of training by the government [113].

## 6. Limitations

Although quite comprehensive, this review has some limitations. No systematic literature search and review was conducted to compile the literature. However, a systematic literature search was not deemed to be a feasible option, considering the numerous diseases as well as the different aspects covered for each disease. The aim of our study was not to provide an overview of all existing literature on the different topics but to provide an overview of the main aspects of important diseases of small ruminants in SSA.

A further limitation stems from the limited availability of literature. Most references described the situation in Ethiopia, whereas no literature on diseases in small ruminants was found for many countries in SSA. Therefore, information on prevalence, economic consequences, and treatment, control, and prevention strategies might not be transferable to all countries in SSA.

Lastly, this review cannot capture the diversity of small ruminant production systems in the various countries in SSA, which would be beyond the scope of this work.

## 7. Conclusions

The diseases included in this review have significant economic implications for small ruminant farmers in SSA. More effective disease control is often hindered by financial constraints. In cases of infection with internal parasites, limited knowledge about the epidemiology of the disease as well as the availability of appropriate anthelmintics and the development of resistance against commonly used anthelmintics are major challenges. The control of viral diseases is dependent on the accessibility, quality, and handling of vaccines, whereas in bacterial diseases, increasing antibiotic resistance and inappropriate antimicrobial treatments are significant obstacles, as well as the availability of appropriate vaccines and their use. In the case of ectoparasitic infections, a strategic, regular, and appropriate antiparasitic treatment approach remains a challenge (Table 5, Table 6, Table 7 and Table 8).

## Figures and Tables

**Table 1:** Recent studies evaluating the anthelmintic resistance of gastrointestinal nematodes against four commonly used anthelmintics in Ethiopia and Zimbabwe, using the percentage fecal egg count reduction test (FECRT).

	FECRT (Lower 95% Confidence Limit)				
	Negash et al., 2023 [20] (Dry Season)	Negash et al., 2023 [20] (Wet Season)	Wondimu and Bayu, 2022 [22]	Solomon et al., 2024 [13]	Mushonga et al., 2024 [21]
	a, b, c	a, b, c	b, d, a	a, b, c	a, b, c
Albendazole	96.0% (84.2%)	97.0% (84.6%)	69.9% (36.9%)	90.4% (82.1%)	70.1% (67.8%)
Tetramisole	97.0% (91.0%)	97.6% (91.0%)	95.7% (87.4%)	96.8% (93.4%)	n.t.
Levamisole	n.t.	n.t.	n.t.	n.t.	85.8% (58.0%)
Ivermectin	98.0% (94.2%)	99.2% (97.8%)	71.1% (38.2%)	92.0% (83.6%)	58.5% (55.4%)

According to the World Association for the Advancement of Veterinary Parasitology, a reduced efficacy, suggesting resistance, is defined as the lower limit of the 95% confidence interval being < 90% [23]. The letters (a–d) refer to the three most frequently isolated nematode genera before treatment: a = *Haemonchus* spp.; b = *Trichostrongylus* spp.; c = *Oesophagostomum* spp.; d = *Teladorsagia* spp. The sequence corresponds to the relative frequency of isolation. n.t. = not tested.

**Table 2:** Percentages of *M. haemolytica* that were resistant against the tested antimicrobials in 3 studies in Ethiopia evaluating the antimicrobial susceptibility patterns of bacteria causing pneumonic pasteurellosis in small ruminants.

	Study		
Antimicrobial	Marru et al., 2013 [98]	Girma et al., 2023 [99]	Abdulkadir et al., 2024 [100]
Ampicillin	46.4%	n.t.	0.0%
Bacitracin	n.t.	83.3%	n.t.
Chloramphenicol	0.0%	0.0%	0.0%
Gentamycin	100%	0.0%	80.0%
Kanamycin	n.t.	n.t.	0.0%
Oxacillin	n.t.	n.t.	80.0%
Penicillin-G	89.3%	50.0%	60.0%
Streptomycin	91.1%	n.t.	n.t.
Sulfamethoxazole	10.7%	0.0%	n.t.
Tetracycline	16.1%	0.0%	20.0%
Vancomycin	100%	n.t.	n.t.

n.t. = not tested.

**Table 3:** Percentages of *P. multocida* that were resistant against the tested antimicrobials in 2 studies in Ethiopia evaluating the antimicrobial susceptibility patterns of bacteria causing pneumonic pasteurellosis in small ruminants.

	Study	
Antimicrobial	Marru et al., 2013 [98]	Abdulkadir et al., 2024 [100]
Ampicillin	50.0%	16.7%
Gentamycin	100%	66.7%
Kanamycin	n.t.	0.0%
Oxacillin	n.t.	100.0%
Penicillin-G	75.0%	66.7%
Streptomycin	87.5%	n.t.
Sulfamethoxazole	12.5%	n.t.
Tetracycline	12.5%	16.7%
Vancomycin	100%	n.t.

n.t. = not tested.

**Table 4:** Prevalence of ectoparasites in sheep/goats in Ethiopia.

		Study			
Ectoparasite	Tesfaye et al., 2012 [115]	Amare et al., 2013 [111]	Seyoum et al., 2015 [114]	Feki et al., 2020 [116]	Jote et al., 2024 [117]
Fleas	13.2%/11.3%	1.1%/2.6%	10.5%/17.7%	0.5%/0.6%	7.9%/7.1%
Ticks	31.4%/12.2%	3.9%/17.7%	22.7%/11.5%	42.7%/33.8%	27.2%/28.0%
Lice	3.8%/9.7%	34.0%/27.8%	23.1%/17.7%	3.2%/1.4%	27.7%/29.6%
Mites	0.0%/0.0%	0.0%/2.2%	0.66%/0.0%	2.7%/3.0%	10.9%/3.0%
*Melophagus ovinus*	1.8%/0.0%	10.8%/0.0%	9.2%/12.5%	0.0%/0.0%	12.4%/0.0%
**Total**	**54.8%/34.9%**	**44.9%/43.5%**	**47.7%/38.5%**	**49.9%**	**58.4%/55.5%**

**Table 5:** Summary of the main economic impacts, current treatment and control strategies of economically important helminthoses in small ruminants, and peculiarities in sub-Saharan Africa (SSA).

Disease	Main Reasons for Economic Losses	Treatment and Control Strategies	Peculiarities in SSA
Gastrointestinal Nematodes	- Productivity losses (weight loss, reduced weight gain, less milk and meat, wool of lower quality) - Mortality	- Anthelmintics - Plants, traditional medicines, and ethnoveterinary remedies - Pasture management	- Development of resistance against anthelmintics - Inappropriate anthelmintic treatment (poor quality drugs, missing knowledge of correct administration, poor dosing, intensive use of a single anthelmintic class)
Lungworms	- Poor body condition, loss of weight or reduced weight gain, unthriftiness - Mortality	- Anthelmintics (albendazole, levamisole, ivermectin) - Improved management praxis (ample nutrition, pasture management, control of snails) - Immunization with live vaccine	- No use of the lungworm vaccine has been reported in Ethiopia
Fasciolosis	- Mortality - Morbidity (reduced growth rate and wool production) - Condemnation of fluky liver	- Anthelmintics (triclabendazole) - Control of snail populations	- Resistance and limited availability of triclabendazole - Unwillingness of farmers to deworm - Missing knowledge (no knowledge of the cause of disease, the importance of snails, and the zoonotic potential)
Cerebral Coenurosis	- Mortality - Lower weight gain - Reduced price of live animals - Head condemnations - Unsuccessful treatment attempts	- Surgery is frequently successful - Anthelmintic treatment (a combination of albendazole, fenbendazole, and praziquantel) is recommended only in very early stages	- Surgery not routinely applicable in SSA - Several frustrating and unsuccessful treatment attempts have been reported - Missing knowledge of the epidemiology of the disease that increases the spread of T. multiceps - No or no effective deworming of dogs

**Table 6:** Summary of the main economic impacts, current treatment and control strategies of economically important viral diseases in small ruminants, and peculiarities in sub-Saharan Africa (SSA).

Disease	Main Reasons for Economic Losses	Treatment and Control Strategies	Peculiarities in SSA
Peste des Petits Ruminants (PPR)	- Mortality - Morbidity	- No specific treatment - Antibiotics (e.g., oxytetracycline) and vitamins - Vaccination	- An international initiative for control of PPR, launched by the FAO and WOAH, aims to eradicate PPR by 2030, relying on vaccination campaigns and disease surveillance
Sheep and Goat Pox	- Mortality - Morbidity (weight loss, decreased milk yield) - Damage to wool and hides - Hindering the introduction of exotic breeds and development of intensive livestock production - Impact on trade, import, and export	- No specific treatment - Antibiotic treatment - Topical treatment - Vaccination	- Vaccine not readily available in all SSA countries - Inadequate infrastructure can hinder mass-vaccination campaigns - Insufficient vaccine quality
Contagious ecthyma (Orf)	- Poor growth of lambs - Mastitis in ewes - Mortality - Hindering the introduction of new breeds and development of intensive animal production	- No specific treatment - Antibiotics in severe cases - Topical antibiotic or antiseptic formulations - Vaccination	- Live attenuated virus and attenuated tissue culture vaccines can disseminate the vaccine strain and are not recommended on farms with no previous history of orf

**Table 7:** Summary of the main economic impacts, current treatment and control strategies of economically important bacterial diseases in small ruminants, and peculiarities in sub-Saharan Africa (SSA).

Disease	Main Reasons for Economic Losses	Treatment and Control Strategies	Peculiarities in SSA
Contagious caprine pleuropneumonia	- Mortality, including abortions - Morbidity (loss of production performance)	- Antimicrobial treatment - Vaccination	- Antibiotic resistance has been identified in some strains - Inappropriate antibiotic treatment without consultation of veterinary professionals - Post-vaccination reactions with an inactivated vaccine produced in Kenya - Lack of technological advances that enable the production of sufficient doses of purified protein vaccine
Pneumonic Pasteurellosis	- High Mortality - High morbidity - High treatment costs - Reduced weight gain, delayed marketing, and unthriftiness	- Antimicrobial treatment - Vaccination	- Resistance against antimicrobials - Vaccines might not match the prevailing serotype
Anthrax	- Mortality - Trade and export restrictions - Treatment costs - Anthrax control measures	- Antimicrobial treatment (e.g., penicillin and oxytetracycline) is successful only in early stages (before production of toxins) - Vaccination	- Antiserum usually not available or used in animals - Vaccination is often neglected - Behaviors such as slaughtering, butchering, and distributing the meat from sick or succumbed animals due to anthrax increase the risk of infections

**Table 8:** Summary of the main economic impacts, current treatment and control strategies of ectoparasite infestations in small ruminants, and peculiarities in sub-Saharan Africa (SSA).

Disease	Main Reasons for Economic Losses	Treatment and Control Strategies	Peculiarities in SSA
Ectoparasite infestations	- Morbidity (loss of production performance) - Rejection or down-graded hide and skin	- Organ phosphorus compounds (e.g., diazinon) - Synthetic pyrethroids (e.g., flumethrin and high cis-cypermethrin) - Macrocyclic lactones (e.g., ivermectin and doramectin)	- Strategic, regular, and appropriate application of the antiparasitic is often not achieved - Prevailing poor veterinary services, improper application of antiparasitics by non-professionals - Absence of environmental control

## Data Availability

Not applicable.
